# Loop2 Size Modification Reveals Significant Impacts on the Potency of α-Conotoxin TxID

**DOI:** 10.3390/md21050286

**Published:** 2023-05-01

**Authors:** Jianying Dong, Panpan Zhang, Junjie Xie, Ting Xie, Xiaopeng Zhu, Dongting Zhangsun, Jinpeng Yu, Sulan Luo

**Affiliations:** 1 School of Medicine, Guangxi University, Nanning 530004, China; 2028301003@st.gxu.edu.cn (J.D.);; 2 Key Laboratory of Tropical Biological Resources, Ministry of Education, Key Laboratory for Marine Drugs of Haikou, Hainan University, Haikou 570228, China

**Keywords:** α-conotoxin TxID, α3β4 nAChR, loop size modification, electrophysiology, CD spectra

## Abstract

α4/6-conotoxin TxID, which was identified from *Conus textile*, simultaneously blocks rat (r) α3β4 and rα6/α3β4 nicotinic acetylcholine receptors (nAChRs) with IC_50_ values of 3.6 nM and 33.9 nM, respectively. In order to identify the effects of loop2 size on the potency of TxID, alanine (Ala) insertion and truncation mutants were designed and synthesized in this study. An electrophysiological assay was used to evaluate the activity of TxID and its loop2-modified mutants. The results showed that the inhibition of 4/7-subfamily mutants [+9A]TxID, [+10A]TxID, [+14A]TxID, and all the 4/5-subfamily mutants against rα3β4 and rα6/α3β4 nAChRs decreased. Overall, ala-insertion or truncation of the 9th, 10th, and 11th amino acid results in a loss of inhibition and the truncation of loop2 has more obvious impacts on its functions. Our findings have strengthened the understanding of α-conotoxin, provided guidance for further modifications, and offered a perspective for future studies on the molecular mechanism of the interaction between α-conotoxins and nAChRs.

## 1. Introduction

Nicotinic acetylcholine receptors (nAChRs) are pentameric ligand-gated ion channels that surround a central water pore [1,2]. They are classified into neuronal nAChRs and muscle-type nAChRs based on their wide distribution in the central and peripheral nervous systems, as well as in non-nervous tissues. Previous studies have shown that nAChRs have 17 distinct subunits: α1–α10, β1–β4, δ, ε, and γ. The α1, β1, δ, ε, and γ subunits form muscle-type nAChRs, whereas the γ subunit is only present in mammalian embryonic cells. The remaining α and β subunits combine to form neuronal nAChR subtypes, including homo-pentamers composed of only α subunits (such as α7 and α9α10 nAChRs) and hetero-pentamers composed of α and β subunits in different ratios (α3β4, α4β2, α6β4 nAChRs, etc.). Each nAChR subunit comprises a certain proportion of various nAChR subtypes with different pharmacological properties [3,4,5]. 

The results of polymerase chain reaction experiments have shown significant RNA expression of α3 and β4 subunits in the human adrenal medulla, human adrenal chromophores, and human cervical squamous carcinoma cells (SiHa) [6,7]. α3β4 nAChR is also significantly expressed in regions of the brain, such as the habenular-internuclear pathway, the hippocampus, the hypothalamus, and the pineal gland [8,9,10]. It is a key regulator of reward-related behaviors in the habenular–internuclear pathway, and is also involved in nicotine-mediated appetite loss in the hypothalamus [11,12,13]. α6-containing (α6*) nAChRs are expressed throughout the peripheral and central nervous systems [14,15,16]. α6β4 nAChR is concentrated in rat dorsal medullary ganglion neurons, which are associated with chronic pain and have become an expected therapeutic target [17,18]. Overall, the distributions of α3β4 and α6β4 nAChRs overlap in some tissues, and the extra-cellular domains (ECDs) of the two receptors are highly homologous. This study will further explain the mechanism of α-conotoxins binding to α3β4 and α6β4 nAChRs and will facilitate the modification and development of targeted drugs.

The cone snails (*Conus*) are a large family of preying gastropods that is currently known to have more than 700 species. Each species of cone snail can secrete 50–200 different kinds of small molecule polypeptides, called conotoxins, in their venom duct, which possess rich pharmacological activities [19,20]. With the improved accuracy of detection technology, it is conservatively estimated that there are over hundreds of thousands of distinct neuroactive conopeptides [21,22]. Among the abundant conotoxins, α-conotoxins from the A-superfamily are the largest subfamily. They are selective and potent antagonists of nAChRs and could be used to distinguish between different nAChR subtypes [23,24,25]. α-conotoxins are usually composed of 12 to 20 amino acids and four cysteines (Cys) arranged in the form of “CC--C--C”, which forms a “globular” pattern (Cys I––III and Cys II–IV). Two pairs of disulfide bonds are found in most native α-conotoxins [26,27]. Based on the number of amino acid residues in the two Cys loops, α-conotoxins can be further classified into 3/5, 4/3, 4/4, 4/5, 4/6, and 4/7 subfamilies [19,28]. 

TxID, an α4/6-conotoxin identified from *Conus textile*, is a competitive antagonist inhibitor with a potent blockade effect on rα3β4 nAChRs, with an IC_50_ value of 3.6 nM. However, TxID also inhibits the rα6/α3β4 nAChR, with an IC_50_ of 33.9 nM [29]. In previous studies, TxID was found to inhibit the growth of human non-small-cell lung cancer A549 and NCI-H1299 cell lines, while displaying an enhanced inhibitory effect when simultaneously treated with adriamycin [30]. TxID was shown to significantly inhibit the proliferation of SiHa [6].

The first Cys loop (loop1) of α-conotoxin contains three amino acids that mainly target muscle-type nAChRs, such as GI, MI, SI, SIA, and SII, whereas α4/7-conotoxins are specific inhibitors of neuronal nAChRs [31,32,33]. The number of amino acids contained in loop2 of α-conotoxins also affects their functions. For example, α4/6-conotoxins AuIB, TxID, and VnIB showed higher selectivity for β4* nAChRs than β2* nAChRs, whereas α4/7-conotoxins MII, OmIA, PIA, TxIB, RegIIA, and PeIA showed higher selectivity for β2* nAChRs [34,35,36,37,38]. Previous findings have shown that a mutation of asparagine (Asn, N) to leucine (Leu, L) at the twelfth position of LsIA enhanced the activity of α3β4 nAChRs [39]. Compared with wild-type RegIIA, [N11A, N12A]RegIIA showed almost a complete loss of potency against human (h) α7, rα3β2, and rα6/α3β4β3 nAChRs, whereas the potency against rα3β4 nAChRs was only decreased 10-fold; thus, [N11A, N12A]RegIIA selectively inhibited rα3β4 nAChRs [40]. Studies of [A10L]PnIA have shown that truncation of the twelfth to fifteenth amino acid residues in loop2 causes [A10L]PnIA to lose its activity against α7 nAChRs [41].

In this study, the amino acids of TxID loop2 were truncated and inserted into alanine (Ala, A), and the activity of TxID mutants was detected using electrophysiology. This will enrich studies on the function of loop2 for α-conotoxins and will also provide a basis for further understandings of the binding mechanism between α4/6-conotoxin TxID and α3β4 nAChRs.

## 2. Results

### 2.1. Synthesis and Characterization of TxID and Its Mutants

**Figure 1:**
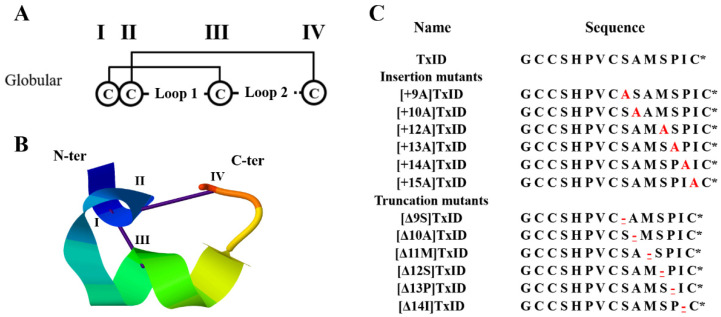
Disulfide bond arrangement and sequences of TxID and its mutations. The disulfide bond arrangement of α-conotoxins is CysI-CysIII and CysII-CysIV, which form two cysteine loops (loop1 and loop2). TxID and its mutants are all globular isomers; “I–IV” indicate the four cysteines in the sequence (**A**). Structure of the globular TxID (PDB: 2m3i); the N- and C-termini are labeled with N-ter and C-ter, respectively (**B**). The inserted alanine is marked red; “-” indicates the truncated amino acid site, which is underlined and marked in red; “*” indicates the C-terminal amide (**C**).

Because the “globular” isomer is the natural and bioactive form of the native TxID, a two-step oxidative folding protocol was used for the oxidation of TxID and all the loop2 size mutants with this disulfide bond arrangement (Figure 1). Afterward, the purity and identity were confirmed by analytical reversed-phase high-performance liquid chromatography (RP-HPLC) and electrospray ionization mass spectroscopy (ESI-MS) (Figures S1 and S2, Table 1). 

### 2.2. Circular Dichroism of TxID and Its Mutants

**Figure 2:**
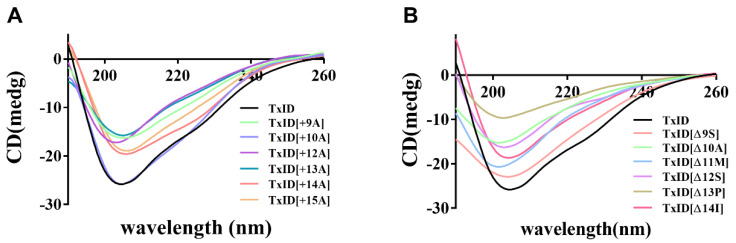
CD spectra of TxID and its insertion (**A**) and truncation mutants (**B**) in H_2_O, *n* = 3. All the peptides have negative peaks at 208 nm and 222 nm, whereas the shapes of the peaks are similar, so the secondary structures of wild-type TxID and its mutants are almost identical.

The circular dichroism (CD) spectra were utilized to analyze whether the loop2 modification had any effect on the secondary structure of TxID. The results indicated that the wild-type (WT) TxID and its mutants showed negative peaks at 208 nm and 222 nm, indicating that all the peptides contain α-helices (Figure 2, Table 2). The percentage of secondary structures were almost identical between the WT-TxID and mutants, with the truncated mutant [Δ13P]TxID showing the largest change. The proportion of α-helices in [Δ13P]TxID decreased by ten percent compared to the native TxID, whereas the proportion of β-sheets increased by twelve percent.

### 2.3. Inhibition of TxID and Its Mutants on rα3β4 and rα6/α3β4 nAChRs

**Figure 3:**
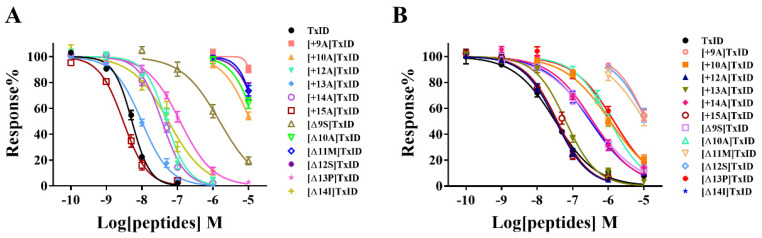
Dose–response curves of wild-type TxID and its mutants on rα3β4 (**A**) and rα6/α3β4 nAChRs (**B**). The dose–response curves of most TxID mutants are right-shifted compared to wild-type TxID, whereas only the curve of [+15A]TxID interacting with rα3β4 nAChRs is slightly left-shifted. Each data point represents the mean ± SEM values from at least 4–10 oocytes.

**Figure 4:**
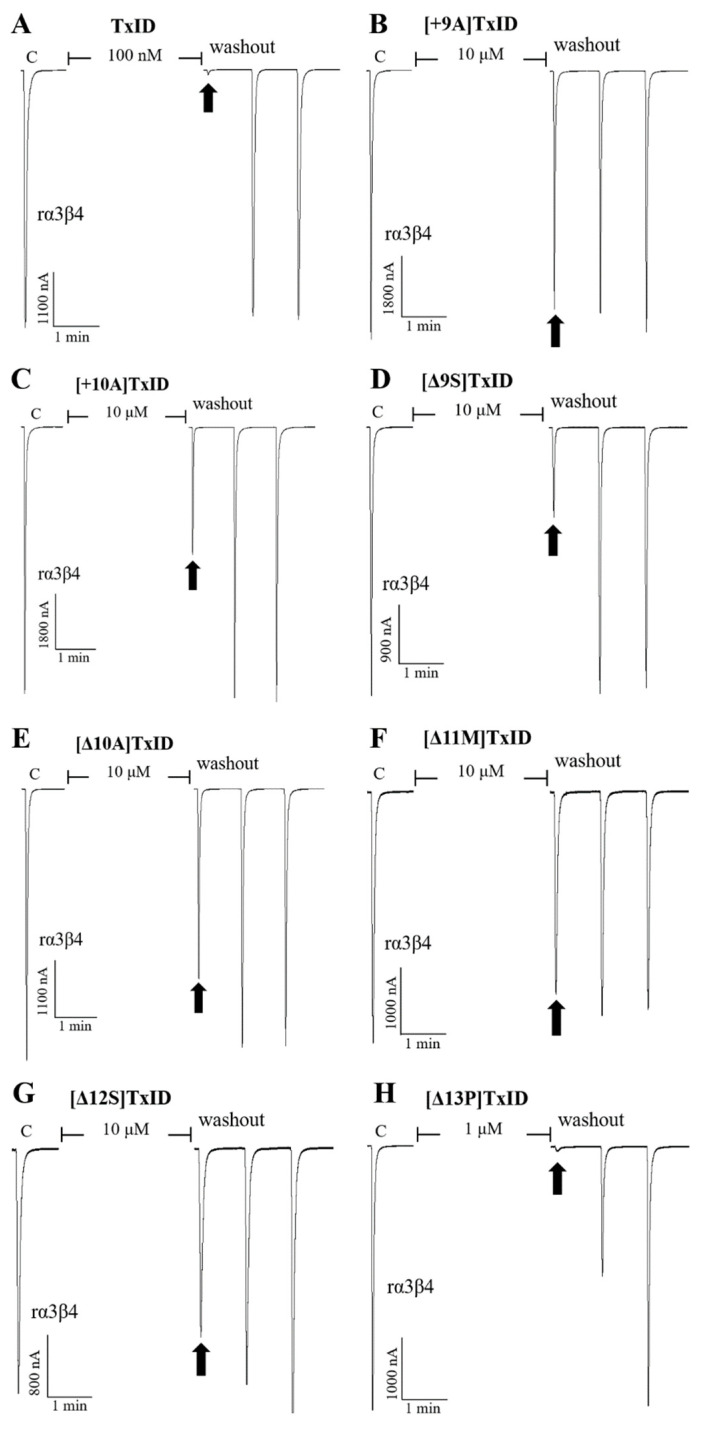
Blocking effects of TxID (**A**) and part of the mutants (**B**–**H**) on rα3β4 nAChRs. Representative responses in individual oocytes are revealed. The arrows indicate the currents generated by 100 μM of ACh stimulation after incubation with different concentrations of α-conotoxins for 5 min. “C” indicates the response to 100 μM of ACh as a control.

**Figure 5:**
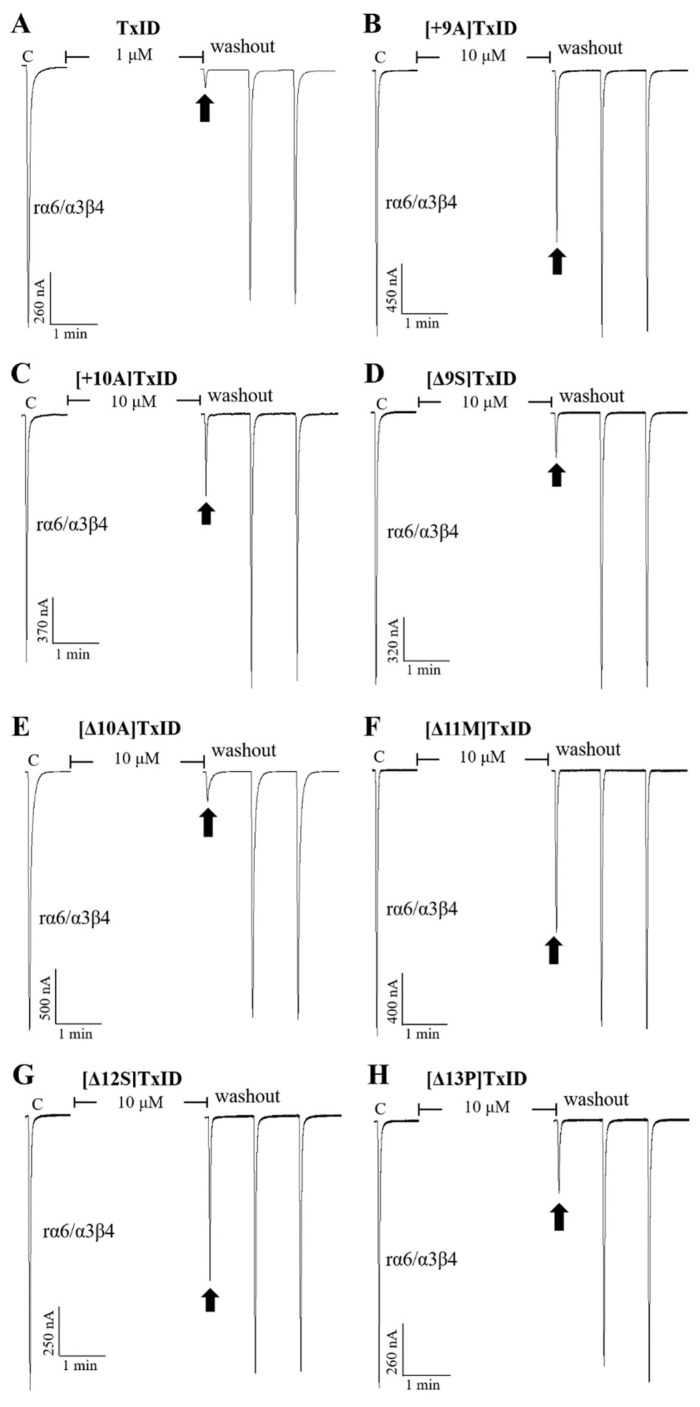
Blocking effects of TxID (**A**) and part of the mutants (**B**–**H**) on rα6/α3β4 nAChRs. Representative responses in individual oocytes are revealed. The arrows indicate the currents generated by 100 μM of ACh stimulation after incubation with different concentrations of α-conotoxins for 5 min. “C” indicates the response to 100 μM of ACh as a control.

The potency of TxID and its mutants on the rα3β4 and rα6/α3β4 nAChR subtypes was evaluated by electrophysiology. The results showed that the IC_50_ values of TxID inhibiting rα3β4 and rα6/α3β4 nAChRs were 5.3 nM and 33 nM, respectively (Figure 3, Figure 4A and Figure 5A, Table 3). 

10 μM [+9A]TxID and [+10A]TxID did not completely inhibit rα3β4 nAChRs, and the blocking percentages were less than fifty percent (Figure 3 and Figure 4B,C, Table 3). The potency of [+12A]TxID and [+14A]TxID against rα3β4 nAChRs decreased 9.2-fold and 7.0-fold, respectively, which are lower potencies compared to that of the native TxID (Figure 3A, Table 3). [+13A]TxID and [+15A]TxID showed similar inhibitory effects of rα3β4 nAChRs, with IC_50_ values of 9.9 nM and 3.0 nM, respectively, which were 1.9-fold and 0.6-fold that of the WT-TxID, respectively (Figure 3A, Table 3). 

The activity assays of loop2 truncation mutants also showed that the 4/5-subfamily mutants [Δ10A]TxID, [Δ11M]TxID, and [Δ12S]TxID exhibited a complete loss of activity against rα3β4 nAChRs (IC_50_ > 10 μM), whereas [Δ9S]TxID exhibited a 292-fold decrease in potency, with an IC_50_ of 1,546 nM (Figure 3A and Figure 4E–G, Table 3). When the truncation sites were closer to the end of the amino acid sequence of TxID, [Δ13P]TxID and [Δ14I]TxID showed a much smaller decrease in activity compared to the other truncation mutants, which showed a 22-fold and 11-fold decrease, with IC_50_ values of 119 nM and 56 nM, respectively (Figure 3A and Figure 4H, Table 3).

The activity of the insertion mutants was also evaluated on rα6/α3β4 nAChRs. The results demonstrated that [+9A]TxID was the only 4/7-subfamily mutant that displayed no obvious inhibition against rα6/α3β4 nAChRs (Figure 3B and Figure 5B, Table 3). The potency of [+10A]TxID and [+14A]TxID decreased 34-fold and 11-fold, respectively, with IC_50_ values of 1130 nM and 368 nM, respectively (Figure 3B and Figure 5C, Table 3). The mutants [+12A]TxID, [+13A]TxID, and [+15A]TxID showed similar activity to that of the WT-TxID (Figure 3B, Table 3). 

When the potencies of the truncation mutants against rα6/α3β4 nAChRs were assessed, [Δ11M]TxID and [Δ12S]TxID showed no significant blocking effect on rα6/α3β4 nAChRs (Figure 3B and Figure 5F–G, Table 3). Other truncation mutants, including [Δ9S]TxID, [Δ10A]TxID, [Δ13P]TxID, and [Δ14I]TxID, displayed different extents of potency decreases that were relative to the native TxID, with IC_50_ values of 351 nM, 1120 nM, 1448 nM, and 316 nM, respectively (Figure 3B and Figure 5D,E,H, Table 3). 

## 3. Discussion

The α3β4 and α6β4 nAChRs are all expressed in both the central and peripheral nervous systems. Hence, they are relevant to many diseases and have become popular targets for drug therapy. However, the ECD of α6β4 nAChRs is highly homologous to that of α3β4 nAChRs, whereas the distribution of these two receptors can overlap in some tissues. Thus, most of the natural α-conotoxins identified to date that selectively target α3β4 nAChRs may also inhibit α6β4 nAChRs, which almost all belong to the 4/7-subfamily and only a few belong to the 4/6-subfamily, including TxID, AuIB, and VnIB (Table 4).

Previous structure–activity relationship (SAR) studies with α-conotoxins have shown that the size of their Cys-loops may affect their pharmacological properties [49,50,51]. Most α-conotoxins have more notable effects on neuronal nAChRs than on muscle-type nAChRs when loop1 contains four amino acids. Some researchers have also demonstrated that the number of amino acids in loop2 affects the activity of α-conotoxins [52]. As shown in Table 4, α4/4-conotoxin BuIA from *C. bullatus* showed insignificant subtype selectivity for nAChRs, although it exhibited high potency against α3β4 nAChRs, with an IC_50_ of 28 nM. Most natural α4/7-conotoxins showed greater inhibitory activity against α6/α3β4 nAChRs than α3β4 nAChRs, such as LvIA from *C. lividus*, PeIA from *C. pergrandis*, and Vc1.1 from *C. victoriae*. In contrast, natural α4/6-conotoxins are more likely to interact with α3β4 nAChRs. For example, AuIB identified from *C. aulicus* could specifically block α3β4 nAChRs with an IC_50_ of 750 nM, without obviously inhibiting other nAChRs. The inhibitory activity of α4/6-conotoxin TxID against α3β4 nAChRs was much higher than that of AuIB, and although it also inhibited α6/α3β4 nAChRs, it apparently had a stronger blocking effect on α3β4 nAChRs. The results in this study showed that the 4/5-subfamily mutants had more severe inhibitory effects against rα3β4 and rα6/α3β4 nAChRs than the 4/7-subfamily mutants, suggesting that the size of loop2 affects the potency of TxID. This may explain why the majority of natural α-conotoxins belonging to the 4/7-subfamily can block α3β4 and α6/α3β4 nAChRs. 

In this study, the loop2 size of α-conotoxin TxID was modified by insertion and truncation, after which the activities of all the mutants were assayed by electrophysiology. As the results showed, when Ser-9, Ala-10, and Met-11 were inserted with alanine or when the original amino acid was truncated, their activities were differentially reduced compared to the wild-type TxID. In previous studies, molecular simulations of TxID binding with α6/α3β4 nAChRs showed that Ser-9 of TxID formed a hydrogen bond with the β4 subunit Lys-81 and was also spatially close to Glu-58, which explains the large loss of activity observed when we inserted Ser-9 [53]. The molecular model of TxID binding with α3β4 nAChRs demonstrated that the deactivation of [+10A]TxID (which is the same as [+11A]TxID) and [Δ11M]TxID was perhaps due to the contact between Met-11 from TxID and Cys-218 from the α3 subunit [53]. Previous studies concerning RegIIA have also shown that β4-K59 and β4-R113 formed hydrogen bonds with residues 9 and 11 of RegIIA, respectively, and that residue 10 formed non-conservative interactions with β4-I111 and β4-L119 [54]. Therefore, we hypothesized that changes to amino acids 9, 10, and 11 on loop2 of the TxID may affect its interactions with rα3β4 nAChRs. 

As described by previous studies, there was a negatively charged Asp residue at position 14 in the α4/6-conotoxin AuIB and a hydrophobic Ile at the corresponding position in the TxID, which may contribute to the different levels of potency and selectivity between AuIB and TxID [29,55]. In our research, the 4/7-subfamily mutant [+14A]TxID showed a greater decrease in activity compared to the other loop2 mutants of backward sites, with a 7.0-fold decrease in activity on rα3β4 and an 11-fold decrease in activity on rα6/α3β4. The NMR structure of TxID showed that one or both of the Pro residues underwent cis-trans isomerization, and the location of Pro-13 in loop2 attracted our attention [29]. Interestingly, the CD results of [+13A]TxID showed no significant changes in its secondary structure. However, a ten percent decrease in α-helices was observed in [Δ13P]TxID, which was the most pronounced change among all the mutants. As the potency of [Δ13P]TxID decreased in the rα3β4 and rα6/α3β4 nAChR activity by 22-fold and 44-fold, respectively, we hypothesized that Pro-13 slightly changed the structure of TxID and thus affected its activity.

Similar half maximal effective concentration (EC_50_) values do not fully represent the same degree of receptor opening, so even though the EC_50_ values of rα3β4 and rα6β4 nAChRs were similar, this study did not compare the IC_50_ values between them [56]. This is very unfortunate for this study, but at the same time, provides a reference for future studies.

In summary, modifications to the loop2 size of α4/6-conotoxin TxID resulting from inserting or truncating individual amino acids affected the activity of TxID targeting α3β4 and α6β4 nAChRs. This provides new insight for the further modification of α-conotoxin TxID and a unique perspective for studying the molecular mechanism of the interaction between α-conotoxins and the related receptors.

## 4. Materials and Methods

### 4.1. Materials and Animals

DH5α competent cells and a plasmid extraction kit were purchased from Vazyme (Nanjing, China). Restriction enzymes, DNA markers, and DNA fragment purification kits were purchased from TaKaRa (Dalian, China). Reversed-phase C18 Vydac columns (5 μm, 4.6 mm × 250 mm; 10 μm, 22 mm × 250 mm) were purchased from Avantor (Radnor, PA, USA). Acetylcholine chloride, atropine, collagenase A, and bovine serum albumin (BSA) were purchased from Sigma (St. Louis, MO, USA). Acetonitrile (ACN, HPLC grade), cRNA mMESSAGE mMACHINE in vitro transcription kits, and RNA MEGA Clear kits were purchased from Thermo Fisher Scientific (Pittsburgh, PA, USA). Trifluoroacetic acid (TFA) was purchased from Aladdin (Shanghai, China). All the other chemical reagents were of analytical grade.

cDNA clones encoding rα3 and rβ4 subunits were kindly provided by S. Heinemann (Salk Institute, San Diego, CA, USA). The rα6/α3 chimera clone was generously provided by J. E. Garrett (Cognetix, Inc., Salt Lake City, UT, USA). Due to the poor expression of the natural rα6 subunit, the rα6/α3 subunit chimera was constructed in previous studies of rα6* nAChR expression in vitro, which includes the ECD of rα6 and the trans-membrane and intra-cellular domains of rα3 [35]. The rβ4 subunit clone in the high expressing pGEMHE vector was generously provided by C. W. Luetje (University of Miami, Miami, FL, USA). 

Female *Xenopus laevis* were obtained from Kunming Institute of Zoology (Kunming, China) and were maintained at 17 °C and fed twice a week for over 6 months before the experiments. 

### 4.2. Peptide Synthesis 

The linear peptides of TxID and its mutants were synthesized by GL Biochemistry (Shanghai, China) using the solid-phase peptide synthesis (SPPS) method. The first and third Cys residues of the peptides were protected in pairs with S-trityl (Trt), whereas the second and fourth were protected with S-acetamidomethyl (Acm). The linear crude peptides were purified using RP-HPLC, and the final active peptides were obtained using a two-step oxidation protocol; all the procedures have been described previously [57,58]. The purification procedure of all linear, monocyclic, and bicyclic peptides was as follows: UV absorption wavelength at 214 nm and a linear gradient of 5–60% solvent B in 55 min at a flow rate of 15 mL/min, where solvent A was ddH_2_O with 0.075% TFA and solvent B was 90% ACN and 10% ddH_2_O with 0.05% TFA. An analytical RP-HPLC was utilized to confirm the purity (>95%) of all the mature peptides. The procedure was as follows: UV absorption wavelength at 214 nm and a linear gradient of 5–50% solvent B in 30 min at a flow rate of 1 mL/min. The molecular masses of the peptides were confirmed by ESI-MS.

### 4.3. Circular Dichroism (CD) Spectra

CD spectra were recorded at room temperature on a Jasco J-810 spectropolarimeter to determine the secondary structure of TxID and its mutants. Each sample was diluted with H_2_O. The test conditions were as follows: a wavelength of 190–260 nm, a scanning rate of 100 nm/min, and a sample cell path length of 1 mm. Each trial had three replicates. The secondary structures of TxID and its mutants were calculated using the CDSSTR method.

### 4.4. cRNA Preparation and Injection 

Plasmids containing cDNA clones of rα3, rα6/α3, and rβ4 nAChR subunits were transformed into the DH5α competent cell for storage and amplification. The cDNAs of the various subunits were linearized and purified with corresponding restriction enzymes, and their products were used as templates to obtain cRNAs by in vitro transcription with T3, T7, and SP6 mMESSAGE mMACHINE transcription kits. The quality and concentration of cRNA were determined by electrophoresis and an ultraviolet spectrophotometer. 

Oocytes were isolated from adult female *Xenopus laevis* and individual oocytes were obtained, as previously described [41]. The animal experiments were approved by the Ethics Committee of Guangxi University, and all the experimental procedures strictly complied with the guidelines for the care and use of laboratory animals. The oocytes were incubated in ND96 solution (96 mM NaCl, 2 mM KCl, 1.8 mM CaCl_2_, 1 mM MgCl_2_, and 5 mM HEPES, pH 7.1–7.5) with antibiotics (10 mg/L penicillin, 10 mg/L streptomycin, and 100 mg /L gentamicin) at 17 °C and at 35% humidity. Glass needles for microinjection were pulled using a P-1000 needle puller (Sutter Instrument Corp., Novato, CA, USA). The cRNAs of different subunits of nAChRs were mixed in equimolar ratios, which were then injected into the oocytes within 24 h of oocyte harvest, ensuring that at least 10 ng of each subunit was injected for each individual oocyte.

### 4.5. Voltage Clamp Recording 

The membrane currents of injected *Xenopus* oocytes were recorded 2-3 days after injection using a two-electrode voltage clamp amplifier (OC-725D, WARNER Instrument, Holliston, MA, USA) and a digital-to-analog converter (Axon 1550B, Molecular Devices, Sunnyvale, CA, USA). The recording electrodes with suitable tips were made from borosilicate glass and had a resistance of 0.5–2 MΩ when filled with 3 M KCl. The oocytes were placed in a cylindrical chamber with a volume of approximately 50 μL and were clamped at a holding potential of −70 mV. During the data recording period, the oocytes were perfused at a flow rate of 2–4 mL/min with ND96 buffer containing 0.1 mg/mL BSA and 1 μM atropine gravitationally, whereas a 2 s pulse of 100 μM ACh was applied per minute to induce the inward current. The oocytes were incubated with ND96 and current values were obtained after ACh stimulation, which were taken as the control responses. The average peak amplitude of the three control responses was used as a baseline. When the TxID and its mutants were incubated, 5 μL of ND96 buffer was removed and replaced with different concentrations of peptides and the currents were recorded after 5 min of incubation. The ratio of ACh-induced current to baseline current after 5 min of incubation with different concentrations of TxID and its mutants allowed us to identify their blocking effects.

### 4.6. Statistical Analysis of Data

GraphPad Prism 7.0 (GraphPad Software, San Diego, CA, USA) was used to fit the data and draw the graphs. The dose–response curves were fitted using non-linear regression according to the following equation: Response% = 100/[1 + ([toxin]/IC_50_)^nH^], where nH represents the Hill coefficient and IC_50_ represents the concentration of the antagonist that produces half-maximal inhibition. All the data points on the dose-response curves were fitted using the mean ± SEM, and each data point was replicated in at least six oocytes, which were obtained from different individual *Xenopus laevis* to ensure the reproducibility and accuracy of the results.

## Figures and Tables

**Table 1:** ESI-MS identified the molecular weight of TxID and its mutants.

Peptides	Retention Time (min)	Elution Concentration of Solvent B *^a^* (%)	Theoretical MW (Da)	Experimental MW (Da)
TxID	15.08	27.6	1489.79	1489.16
Insertion mutants				
[+9A]TxID	20.10	35.2	1560.87	1560.78
[+10A]TxID	16.68	30.0	1560.87	1560.64
[+12A]TxID	16.72	30.1	1560.87	1560.50
[+13A]TxID	15.79	28.7	1560.87	1560.38
[+14A]TxID	17.00	30.5	1560.87	1560.78
[+15A]TxID	17.19	30.8	1560.87	1560.9
Truncation mutants				
[Δ9S]TxID	15.46	28.2	1402.72	1402.12
[Δ10A]TxID	20.82	36.2	1418.72	1418.62
[Δ11M]TxID	17.75	31.6	1358.60	1358.56
[Δ12S]TxID	16.78	30.2	1402.72	1402.52
[Δ13P]TxID	18.12	32.2	1392.68	1392.22
[Δ14I]TxID	15.57	28.4	1376.64	1376.66

*^a^* Solvent B is 90% ACN and 10% ddH_2_O with 0.05% TFA.

**Table 2:** Secondary structure percentages of the native TxID and its mutants.

Peptides	α-Helix (%)	β-Sheet (%)	β-Turn (%)	Random Coil (%)
TxID	19%	21%	27%	33%
[+9A]TxID	14%	26%	25%	35%
[+10A]TxID	20%	23%	27%	29%
[+12A]TxID	12%	28%	24%	36%
[+13A]TxID	15%	25%	25%	35%
[+14A]TxID	19%	20%	25%	35%
[+15A]TxID	17%	24%	26%	33%
[Δ9S]TxID	14%	32%	26%	28%
[Δ10A]TxID	11%	30%	25%	34%
[Δ11M]TxID	14%	27%	26%	34%
[Δ12S]TxID	13%	27%	26%	34%
[Δ13P]TxID	9%	33%	24%	33%
[Δ14I]TxID	12%	29%	24%	34%

**Table 3:** The potency and selectivity of the native TxID and its mutants against rα3β4 and rα6/α3β4 nAChRs.

WT and Mutant Peptides	rα3β4		Ratio Relative to TxID *^b^*	rα6/α3β4		Ratio Relative to TxID *^b^*
	IC_50_ (95% CI) *^a^* (nM)	Hill Slope		IC_50_ (95% CI) *^a^* (nM)	Hill Slope	
TxID	5.3 (4.8–5.7)	1.7 (1.4–2.1)	1.0	33 (28–38)	0.8 (0.7–0.9)	1.0
Insertion mutants						
[+9A]TxID	>10,000 ^*c*^	/	/	>10,000 ^*c*^	/	/
[+10A]TxID	>10,000 *^c^*	/	/	1130 (873–1480)	0.7 (0.6–0.8)	34
[+12A]TxID	49 (42–57)	1.4 (1.1–2.2)	9.2	34 (28–40)	1.0 (0.8–1.1)	1.0
[+13A]TxID	9.9 (8.6–11)	1.1 (1.0–1.3)	1.9	64 (53–77)	1.0 (0.8–1.2)	2.0
[+14A]TxID	37 (32–42)	1.3 (1.1–1.6)	7.0	368 (271–504)	0.7 (0.6–0.9)	11
[+15A]TxID	3.0 (2.6–3.5)	1.3 (1.1–1.6)	0.6	37 (31–45)	1.0 (0.8–1.1)	1.1
Truncation mutants						
[Δ9S]TxID	1546 (1079–2275)	0.8 (0.6–1.1)	292	351 (271–458)	0.6 (0.5–0.7)	11
[Δ10A]TxID	>10,000 ^*c*^	/	/	1120 (899–1437)	0.9 (0.7–1.1)	34
[Δ11M]TxID	>10,000 ^*c*^	/	/	>10,000 ^*c*^	/	/
[Δ12S]TxID	>10,000 ^*c*^	/	/	>10,000 ^*c*^	/	/
[Δ13P]TxID	119 (99–146)	0.9 (0.8–1.1)	22	1448 (1092–1911)	0.8 (0.6–0.9)	44
[Δ14I]TxID	56 (44–72)	0.8 (0.7–1.1)	11	316 (243–410)	0.7 (0.6–0.8)	9.6

*^a^* IC_50_ values are reported with 95% confidence intervals. *^b^* Ratio of mutant TxID to wild-type TxID IC_50_ values. *^c^* The current responses induced by 100 μM of acetylcholine (ACh) are >50% after incubation with 10 μM of peptides.

**Table 4:** Native α-conotoxins that target the α3β4 and α6β4 nAChRs.

Name	*Conus*	Subfamily	Sequence	IC_50_ on α3β4 nAChR (nM)	Selectivity on nAChRs	Ref.
TxID	*C. textile*	4/6	GCCSHPVCSAMSPIC*	3.6	α3β4 > α6/α3β4 > α2β4	[29]
AuIB	*C. aulicus*	4/6	GCCSYPPCFATNPDC*	750	α3β4	[42]
VnIB	*C. ventricosus*	4/6	GGCCSHPVCYTKNPNCG*	360	α6/α3β4 > α3β4 > α6/α3β2β3	[43]
BuIA	*C. bullatus*	4/4	GCCSTPPCAVLYC*	27.7	α6/α3β2β3 > α6/α3β4 > α3β2 > α3β4 > α4β4 > α7 > α2β2 = α2β4	[44,45]
LvIA	*C. lividus*	4/7	GCCSHPACNVDHPEIC*	148	α3β2 > α6/α3β2β3 > α6/α3β4 > α3β4 > α7	[46]
PeIA	*C. pergrandis*	4/7	GCCSHPACSVNHPELC*	480	α9α10 > α6/α3β2β3 > α3β2 > α6/α3β4 > α3β4 > α7	[38,47]
PIA	*C. purpurascens*	4/7	RDPCCSNPVCTVHNPQIC*	518	α6/α3β2β3 > α6/α3β4 > α3β2 > α3β4	[35]
RegIIA	*C. regius*	4/7	GCCSHPACNVNNPHIC*	97	α3β2 > α3β4 > α7	[37]
Vc1.1	*C. victoriae*	4/7	GCCSDPRCNYDHPEIC*	4,200	α9α10 > α6/α3β2β3 > α6/α3β4 > α3β4 > α3β2	[48]

* indicates a C-terminal amide.

## Data Availability

The data presented in this study are available in the article.

## Supplementary Materials

The following supporting information can be downloaded at: https://www.mdpi.com/article/10.3390/md21050286/s1, additional figures illustrating the RP-HPLC and ESI-MS analysis of TxID and its mutants (Figures S1 and S2).

## Abbreviations Used

ACh, acetylcholine chloride; ACN, acetonitrile; Acm, S-acetamidomethyl; BSA, bovine serum albumin; CI, confidence interval; ESI-MS, electrospray ionization mass spectroscopy; IC_50_, half maximal inhibitory concentration; K_3_[Fe(CN)_6_], potassium ferricyanide; MW, molecular mass; nAChRs, nicotinic acetylcholine receptors; RP-HPLC, reversed-phase high-performance liquid chromatography; SPPS, solid-phase peptide synthesis; Trt, S-trityl; TFA, trifluoroacetic acid.

## References

[1] Corradi J., Bouzat C. (2016). Understanding the Bases of Function and Modulation of alpha7 Nicotinic Receptors: Implications for Drug Discovery. Mol. Pharmacol..

[2] Olivera B.M., Quik M., Vincler M., McIntosh J.M. (2008). Subtype-selective conopeptides targeted to nicotinic receptors: Concerted discovery and biomedical applications. Channels.

[3] Gharpure A., Noviello C.M., Hibbs R.E. (2020). Progress in nicotinic receptor structural biology. Neuropharmacology.

[4] Ho T.N.T., Abraham N., Lewis R.J. (2020). Structure-Function of Neuronal Nicotinic Acetylcholine Receptor Inhibitors Derived From Natural Toxins. Front. Neurosci..

[5] Smith N.J., Hone A.J., Memon T., Bossi S., Smith T.E., McIntosh J.M., Olivera B.M., Teichert R.W. (2013). Comparative functional expression of nAChR subtypes in rodent DRG neurons. Front. Cell Neurosci..

[6] Liu Y., Qian J., Sun Z., Zhangsun D., Luo S. (2019). Cervical Cancer Correlates with the Differential Expression of Nicotinic Acetylcholine Receptors and Reveals Therapeutic Targets. Mar. Drugs..

[7] Hone A.J., McIntosh J.M., Azam L., Lindstrom J., Lucero L., Whiteaker P., Passas J., Blazquez J., Albillos A. (2015). alpha-Conotoxins Identify the alpha3beta4* Subtype as the Predominant Nicotinic Acetylcholine Receptor Expressed in Human Adrenal Chromaffin Cells. Mol. Pharmacol..

[8] Rivera-Perez L.M., Kwapiszewski J.T., Roberts M.T. (2021). alpha(3)beta(4)(*) Nicotinic Acetylcholine Receptors Strongly Modulate the Excitability of VIP Neurons in the Mouse Inferior Colliculus. Front. Neural. Circuits.

[9] Grady S.R., Moretti M., Zoli M., Marks M.J., Zanardi A., Pucci L., Clementi F., Gotti C. (2009). Rodent habenulo-interpeduncular pathway expresses a large variety of uncommon nAChR subtypes, but only the alpha3beta4* and alpha3beta3beta4* subtypes mediate acetylcholine release. J. Neurosci..

[10] Cao Y.J., Surowy C.S., Puttfarcken P.S. (2005). Nicotinic acetylcholine receptor-mediated [3H]dopamine release from hippocampus. J. Pharmacol. Exp. Ther..

[11] Yuan M., Malagon A.M., Yasuda D., Belluzzi J.D., Leslie F.M., Zaveri N.T. (2017). The alpha3beta4 nAChR partial agonist AT-1001 attenuates stress-induced reinstatement of nicotine seeking in a rat model of relapse and induces minimal withdrawal in dependent rats. Behav. Brain Res..

[12] Stoker A.K., Markou A. (2013). Unraveling the neurobiology of nicotine dependence using genetically engineered mice. Curr. Opin. Neurobiol..

[13] Wills L., Ables J.L., Braunscheidel K.M., Caligiuri S.P.B., Elayouby K.S., Fillinger C., Ishikawa M., Moen J.K., Kenny P.J. (2022). Neurobiological Mechanisms of Nicotine Reward and Aversion. Pharmacol. Rev..

[14] Letchworth S.R., Whiteaker P. (2011). Progress and challenges in the study of alpha6-containing nicotinic acetylcholine receptors. Biochem. Pharmacol..

[15] Azam L., McIntosh J.M. (2006). Characterization of nicotinic acetylcholine receptors that modulate nicotine-evoked [3H]norepinephrine release from mouse hippocampal synaptosomes. Mol. Pharmacol..

[16] Quik M., Perez X.A., Grady S.R. (2011). Role of α6 nicotinic receptors in CNS dopaminergic function: Relevance to addiction and neurological disorders. Biochem. Pharmacol..

[17] Hone A.J., Meyer E.L., McIntyre M., McIntosh J.M. (2012). Nicotinic acetylcholine receptors in dorsal root ganglion neurons include the α6β4 subtype. FASEB J..

[18] Wieskopf J.S., Mathur J., Limapichat W., Post M.R., Al-Qazzaz M., Sorge R.E., Martin L.J., Zaykin D.V., Smith S.B., Freitas K. (2015). The nicotinic alpha6 subunit gene determines variability in chronic pain sensitivity via cross-inhibition of P2X2/3 receptors. Sci. Transl. Med..

[19] Lebbe E.K., Peigneur S., Wijesekara I., Tytgat J. (2014). Conotoxins targeting nicotinic acetylcholine receptors: An overview. Mar. Drugs.

[20] Sanchez-Campos N., Bernaldez-Sarabia J., Licea-Navarro A.F. (2022). Conotoxin Patenting Trends in Academia and Industry. Mar. Drugs.

[21] Akondi K.B., Muttenthaler M., Dutertre S., Kaas Q., Craik D.J., Lewis R.J., Alewood P.F. (2014). Discovery, synthesis, and structure-activity relationships of conotoxins. Chem. Rev..

[22] Margiotta F., Micheli L., Ciampi C., Ghelardini C., McIntosh J.M., Di Cesare Mannelli L. (2022). Conus regius-Derived Conotoxins: Novel Therapeutic Opportunities from a Marine Organism. Mar. Drugs..

[23] Nicke A., Wonnacott S., Lewis R.J. (2004). Alpha-conotoxins as tools for the elucidation of structure and function of neuronal nicotinic acetylcholine receptor subtypes. Eur. J. Biochem..

[24] Azam L., McIntosh J.M. (2009). Alpha-conotoxins as pharmacological probes of nicotinic acetylcholine receptors. Acta Pharmacol. Sin..

[25] Daly J.W. (2005). Nicotinic agonists, antagonists, and modulators from natural sources. Cell Mol. Neurobiol..

[26] Kaas Q., Westermann J.C., Craik D.J. (2010). Conopeptide characterization and classifications: An analysis using ConoServer. Toxicon.

[27] Ma Q., Tae H.S., Wu G., Jiang T., Yu R. (2017). Exploring the Relationship between Nicotinic Acetylcholine Receptor Ligand Size, Efficiency, Efficacy, and C-Loop Opening. J. Chem. Inf. Model.

[28] Jin A.H., Muttenthaler M., Dutertre S., Himaya S.W.A., Kaas Q., Craik D.J., Lewis R.J., Alewood P.F. (2019). Conotoxins: Chemistry and Biology. Chem. Rev..

[29] Luo S., Zhangsun D., Zhu X., Wu Y., Hu Y., Christensen S., Harvey P.J., Akcan M., Craik D.J., McIntosh J.M. (2013). Characterization of a novel alpha-conotoxin TxID from Conus textile that potently blocks rat alpha3beta4 nicotinic acetylcholine receptors. J. Med. Chem..

[30] Qian J., Liu Y.Q., Sun Z.H., Zhangsun D.T., Luo S.L. (2019). Identification of nicotinic acetylcholine receptor subunits in different lung cancer cell lines and the inhibitory effect of alpha-conotoxin TxID on lung cancer cell growth. Eur. J. Pharmacol..

[31] Gehrmann J., Alewood P.F., Craik D.J. (1998). Structure determination of the three disulfide bond isomers of α-conotoxin GI: A model for the role of disulfide bonds in structural stability11Edited by P. E. Wright. J. Mol. Biol..

[32] Wilhelm P., Luna-Ramirez K., Chin Y.K.Y., Dekan Z., Abraham N., Tae H.-S., Chow C.Y., Eagles D.A., King G.F., Lewis R.J. (2022). Cysteine-Rich α-Conotoxin SII Displays Novel Interactions at the Muscle Nicotinic Acetylcholine Receptor. ACS Chem. Neurosci..

[33] Guo H., Deng B., Zhao L., Gao Y., Zhang X., Yang C., Zou B., Chen H., Sun M., Wang L. (2022). Programmed Aptamer Screening, Characterization, and Rapid Detection for alpha-Conotoxin MI. Toxins.

[34] Chi S.W., Kim D.H., Olivera B.M., McIntosh J.M., Han K.H. (2006). Solution conformation of a neuronal nicotinic acetylcholine receptor antagonist alpha-conotoxin OmIA that discriminates alpha3 vs. alpha6 nAChR subtypes. Biochem. Biophys Res. Commun..

[35] Dowell C., Olivera B.M., Garrett J.E., Staheli S.T., Watkins M., Kuryatov A., Yoshikami D., Lindstrom J.M., McIntosh J.M. (2003). α-Conotoxin PIA Is Selective for α6 Subunit-Containing Nicotinic Acetylcholine Receptors. J. Neurosci..

[36] Spira M.E., Hasson A., Fainzilber M., Gordon D., Zlotkin E. (1993). Chemical and electrophysiological characterization of new peptide neurotoxins from the venom of the molluscivorous snail Conus textile neovicarius: A review. Isr. J. Med. Sci..

[37] Franco A., Kompella S.N., Akondi K.B., Melaun C., Daly N.L., Luetje C.W., Alewood P.F., Craik D.J., Adams D.J., Mari F. (2012). RegIIA: An alpha4/7-conotoxin from the venom of Conus regius that potently blocks alpha3beta4 nAChRs. Biochem. Pharmacol..

[38] McIntosh J.M., Plazas P.V., Watkins M., Gomez-Casati M.E., Olivera B.M., Elgoyhen A.B. (2005). A novel alpha-conotoxin, PeIA, cloned from Conus pergrandis, discriminates between rat alpha9alpha10 and alpha7 nicotinic cholinergic receptors. J. Biol. Chem..

[39] Inserra M.C., Kompella S.N., Vetter I., Brust A., Daly N.L., Cuny H., Craik D.J., Alewood P.F., Adams D.J., Lewis R.J. (2013). Isolation and characterization of alpha-conotoxin LsIA with potent activity at nicotinic acetylcholine receptors. Biochem. Pharmacol..

[40] Kompella S.N., Hung A., Clark R.J., Mari F., Adams D.J. (2015). Alanine scan of alpha-conotoxin RegIIA reveals a selective alpha3beta4 nicotinic acetylcholine receptor antagonist. J. Biol. Chem..

[41] Hogg R.C., Hopping G., Alewood P.F., Adams D.J., Bertrand D. (2003). Alpha-conotoxins PnIA and [A10L]PnIA stabilize different states of the alpha7-L247T nicotinic acetylcholine receptor. J. Biol. Chem..

[42] Luo S., Kulak J.M., Cartier G.E., Jacobsen R.B., Yoshikami D., Olivera B.M., McIntosh J.M. (1998). α-Conotoxin AuIB Selectively Blocks α3β4 Nicotinic Acetylcholine Receptors and Nicotine-Evoked Norepinephrine Release. J. Neurosci..

[43] van Hout M., Valdes A., Christensen S.B., Tran P.T., Watkins M., Gajewiak J., Jensen A.A., Olivera B.M., McIntosh J.M. (2019). α-Conotoxin VnIB from Conus ventricosus is a potent and selective antagonist of α6β4* nicotinic acetylcholine receptors. Neuropharmacology.

[44] Azam L., Dowell C., Watkins M., Stitzel J.A., Olivera B.M., McIntosh J.M. (2005). Alpha-conotoxin BuIA, a novel peptide from Conus bullatus, distinguishes among neuronal nicotinic acetylcholine receptors. J. Biol. Chem..

[45] Azam L., Maskos U., Changeux J.P., Dowell C.D., Christensen S., De Biasi M., McIntosh J.M. (2010). alpha-Conotoxin BuIA[T5A;P6O]: A novel ligand that discriminates between alpha6ss4 and alpha6ss2 nicotinic acetylcholine receptors and blocks nicotine-stimulated norepinephrine release. FASEB J..

[46] Luo S., Zhangsun D., Schroeder C.I., Zhu X., Hu Y., Wu Y., Weltzin M.M., Eberhard S., Kaas Q., Craik D.J. (2014). A novel alpha4/7-conotoxin LvIA from Conus lividus that selectively blocks alpha3beta2 vs. alpha6/alpha3beta2beta3 nicotinic acetylcholine receptors. FASEB J..

[47] Hone A.J., Ruiz M., Scadden M., Christensen S., Gajewiak J., Azam L., McIntosh J.M. (2013). Positional scanning mutagenesis of alpha-conotoxin PeIA identifies critical residues that confer potency and selectivity for alpha6/alpha3beta2beta3 and alpha3beta2 nicotinic acetylcholine receptors. J. Biol. Chem..

[48] Vincler M., Wittenauer S., Parker R., Ellison M., Olivera B.M., McIntosh J.M. (2006). Molecular mechanism for analgesia involving specific antagonism of α9α10 nicotinic acetylcholine receptors. Proc. Natl. Acad. Sci. USA.

[49] Turner M., Eidemiller S., Martin B., Narver A., Marshall J., Zemp L., Cornell K.A., McIntosh J.M., McDougal O.M. (2009). Structural basis for alpha-conotoxin potency and selectivity. Bioorg. Med. Chem..

[50] Loughnan M.L., Alewood P.F. (2004). Physico-chemical characterization and synthesis of neuronally active alpha-conotoxins. Eur. J. Biochem..

[51] Craig A.G., Bandyopadhyay P., Olivera B.M. (1999). Post-translationally modified neuropeptides from Conus venoms. Eur. J. Biochem..

[52] Giribaldi J., Dutertre S. (2018). alpha-Conotoxins to explore the molecular, physiological and pathophysiological functions of neuronal nicotinic acetylcholine receptors. Neurosci. Lett..

[53] Wu Y., Zhangsun D., Zhu X., Kaas Q., Zhangsun M., Harvey P.J., Craik D.J., McIntosh J.M., Luo S. (2017). alpha-Conotoxin [S9A]TxID Potently Discriminates between alpha3beta4 and alpha6/alpha3beta4 Nicotinic Acetylcholine Receptors. J. Med. Chem..

[54] Xu Q., Tae H.S., Wang Z., Jiang T., Adams D.J., Yu R. (2020). Rational Design of alpha-Conotoxin RegIIA Analogues Selectively Inhibiting the Human alpha3beta2 Nicotinic Acetylcholine Receptor through Computational Scanning. ACS Chem. Neurosci..

[55] Lee B.H., Hwang S.H., Choi S.H., Shin T.J., Kang J., Lee S.M., Nah S.Y. (2011). Quercetin Inhibits alpha3beta4 Nicotinic Acetylcholine Receptor-Mediated Ion Currents Expressed in Xenopus Oocytes. Korean J. Physiol. Pharmacol..

[56] Germann A.L., Pierce S.R., Evers A.S., Steinbach J.H., Akk G. (2022). Perspective on the Relationship between GABAA Receptor Activity and the Apparent Potency of an Inhibitor. Curr. Neuropharmacol..

[57] Fuller E., Green B.R., Catlin P., Buczek O., Nielsen J.S., Olivera B.M., Bulaj G. (2005). Oxidative folding of conotoxins sharing an identical disulfide bridging framework. FEBS J..

[58] Steiner A.M., Bulaj G. (2011). Optimization of oxidative folding methods for cysteine-rich peptides: A study of conotoxins containing three disulfide bridges. J. Pept. Sci..

