# Supplementary material for: Loop2 Size Modification Reveals Significant Impacts on the Potency of α-Conotoxin TxID
Source: Mar Drugs. 2023 May 1;21(5):286. doi: 10.3390/md21050286 (PMC10222858; doi:10.3390/md21050286)
Supplement: Supplementary file 1 [file marinedrugs-21-00286-s001.zip › marinedrugs-2357576-supplementary.pdf]

Supporting Information

Loop2 Size Modification Reveals Significant Impacts on the Potency of α-Conotoxin TxID

Jianying Dong[a], Panpan Zhang[a], Junjie Xie[a], Ting Xie[a], Xiaopeng Zhu[a], Dongting Zhangsun[b], Jinpeng Yu[a]*, Sulan Luo[a, b]*

[a] School of Medicine, Guangxi University, Nanning, 530004, China

[b] Key Laboratory of Tropical Biological Resources, Ministry of Education; Key Laboratory for Marine Drugs of Haikou, Hainan University, Haikou 570228, China

* Corresponding Author E-mail: yujinpeng@gxu.edu.cn (J.Y.); Sulan2021@gxu.edu.cn (S.L.);

Table of Contents

Figure S1. RP-HPLC analysis of TxID and its mutants. (Page S2-S3)

Figure S2. ESI-MS analysis of TxID and its mutants. (Pages S4-S5)

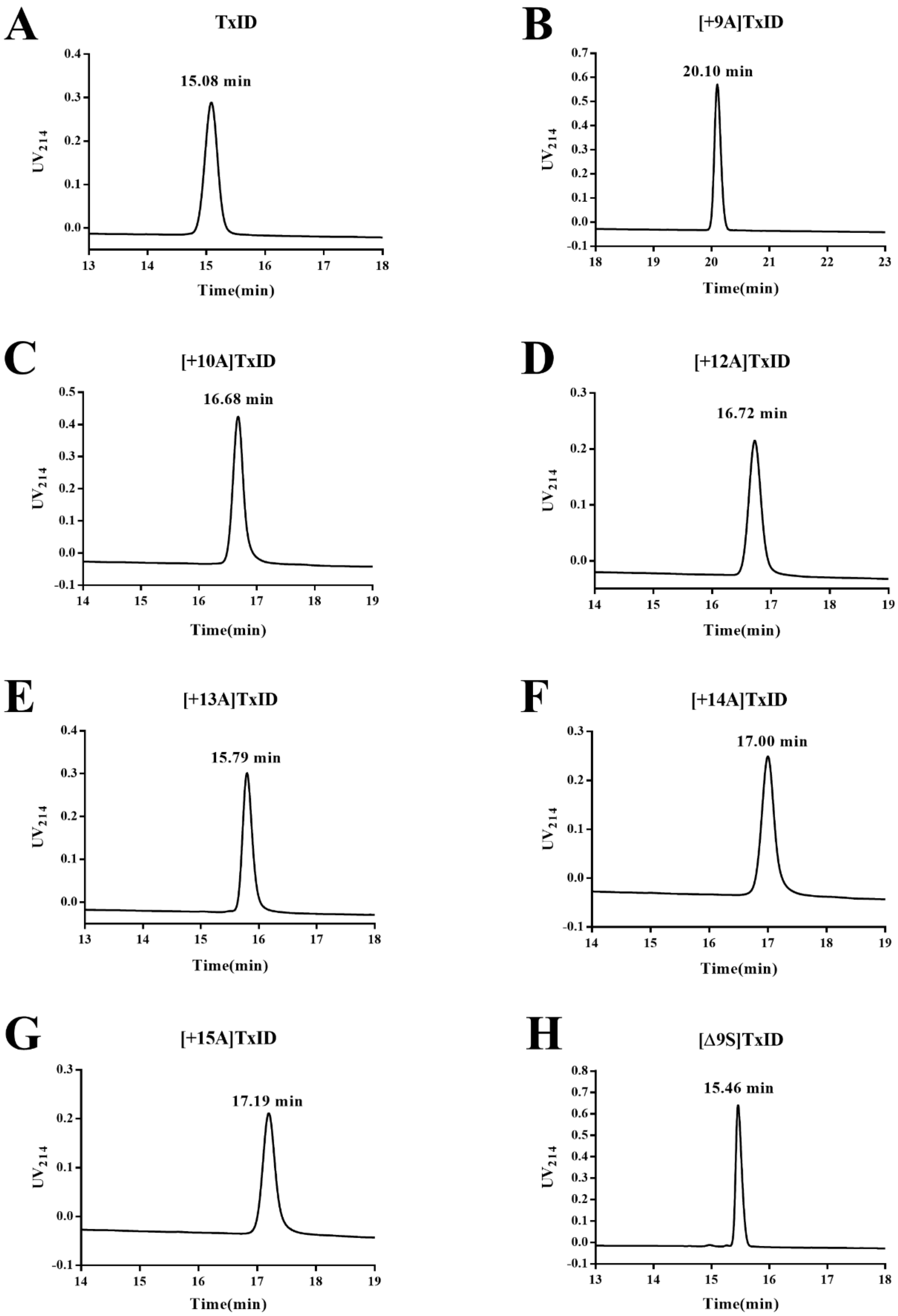
A
TxID
15.08 min
B
[+9A]TxID
20.10 min
C
[+10A]TxID
16.68 min
D
[+12A]TxID
16.72 min
E
[+13A]TxID
15.79 min
F
[+14A]TxID
17.00 min
G
[+15A]TxID
17.19 min
H
[Δ9S]TxID
15.46 min
UV_{214}
Time(min)

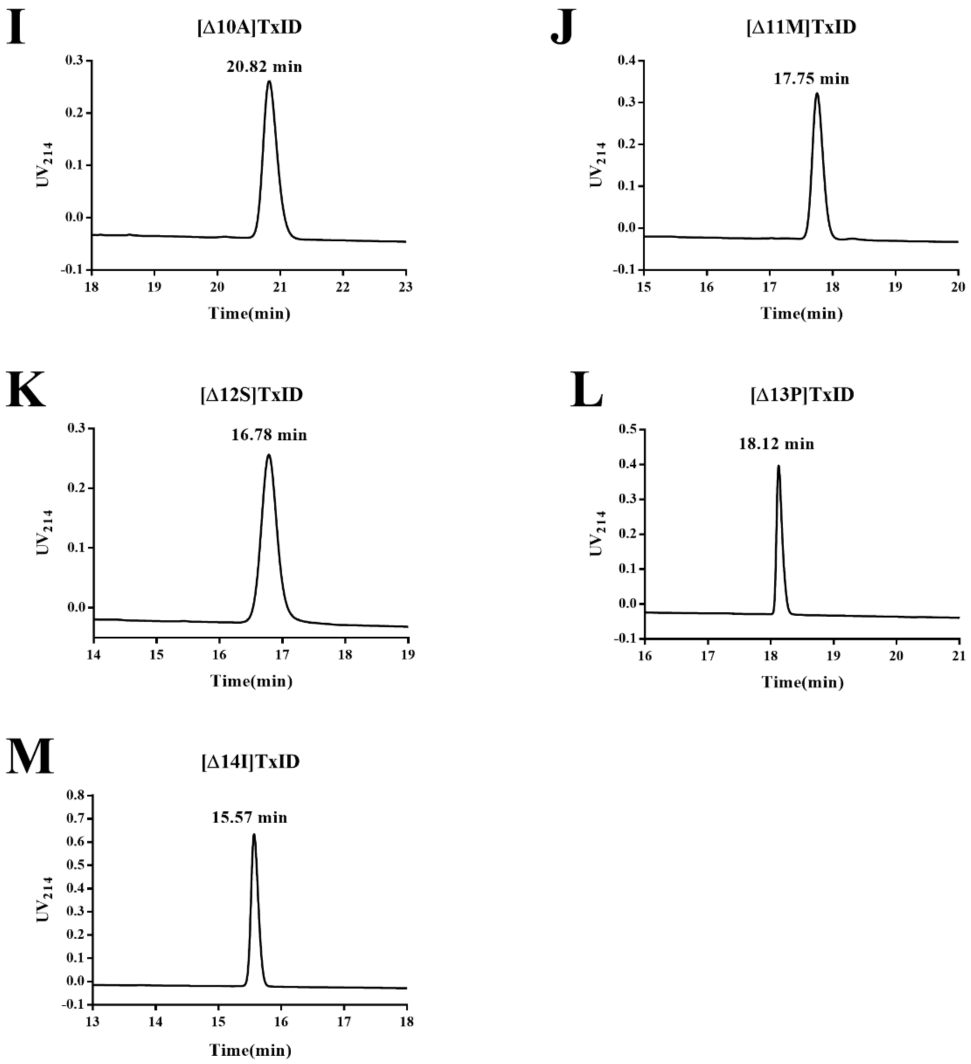

Figure S1. RP-HPLC analysis of TxID and its mutants (A-M).

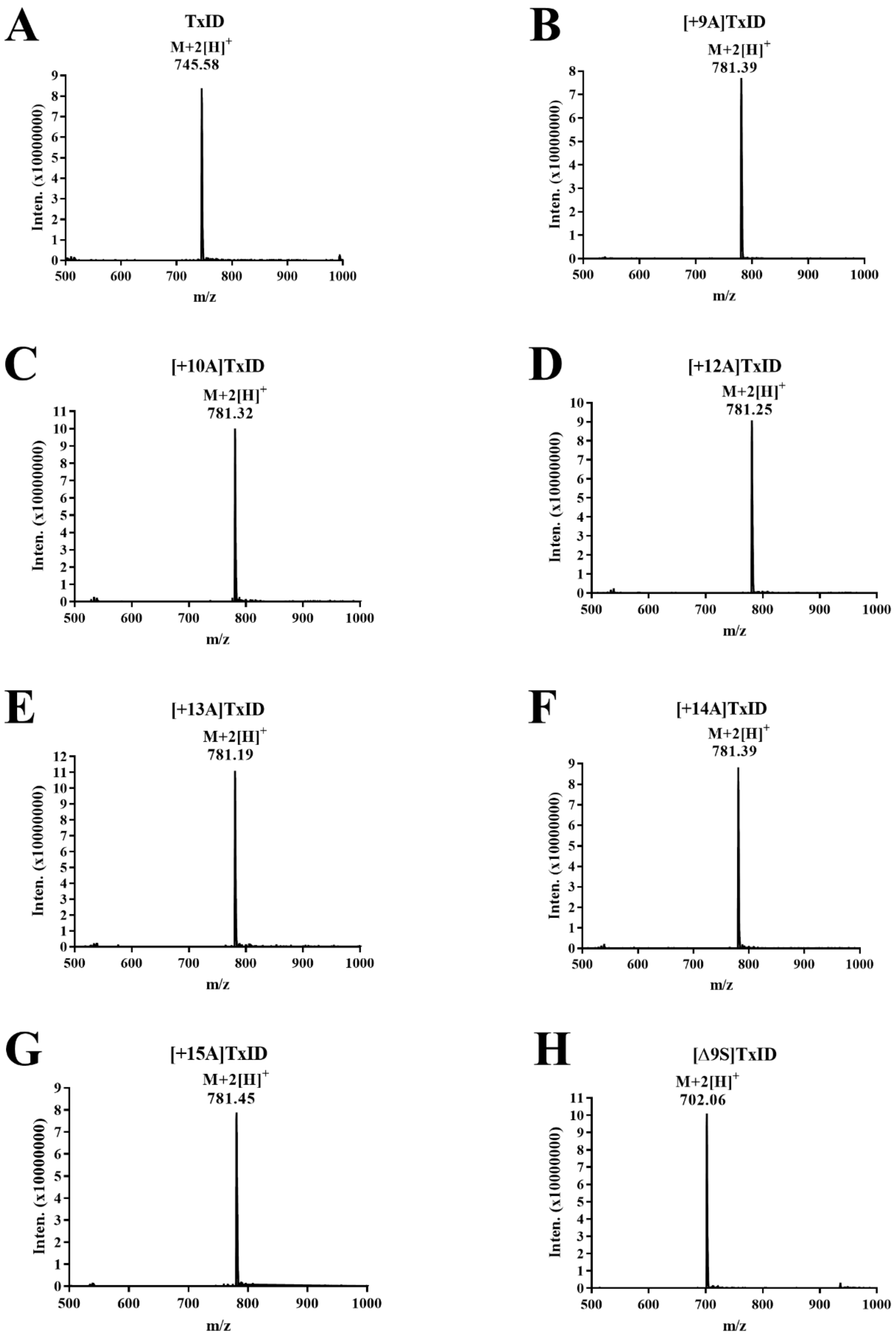
A
TxID
M+2[H]+
745.58
B
[+9A]TxID
M+2[H]+
781.39
C
[+10A]TxID
M+2[H]+
781.32
D
[+12A]TxID
M+2[H]+
781.25
E
[+13A]TxID
M+2[H]+
781.19
F
[+14A]TxID
M+2[H]+
781.39
G
[+15A]TxID
M+2[H]+
781.45
H
[Δ9S]TxID
M+2[H]+
702.06
Inten. (x10000000)
m/z

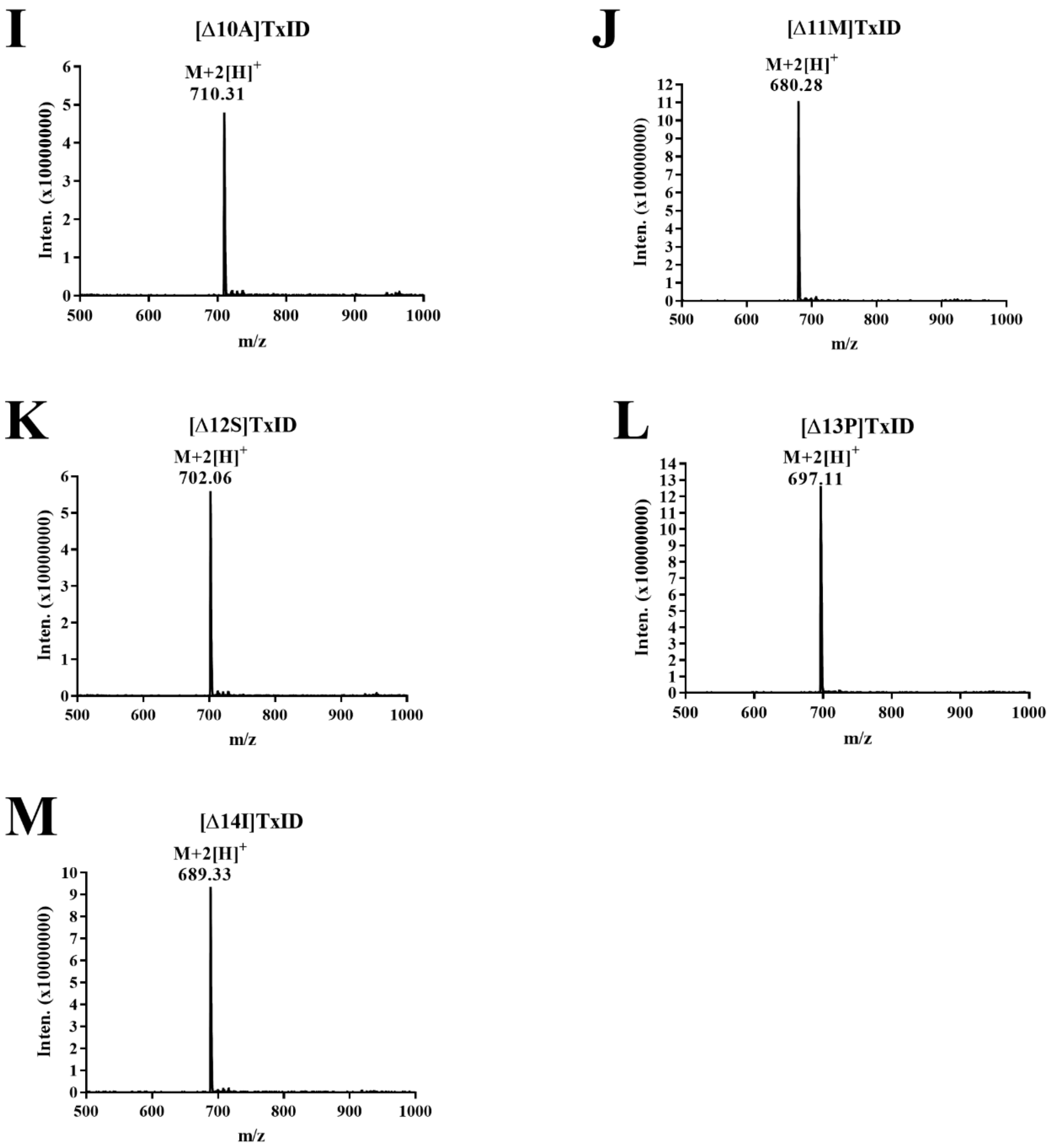

Figure S2. ESI-MS analysis of TxID and its mutants (A-M).
